# Editorial: Innovative AI approaches in quantitative MRI: from image enhancement to biomarker estimation

**DOI:** 10.3389/fradi.2026.1796451

**Published:** 2026-02-16

**Authors:** Alfonso Mastropietro, Elisa Scalco, Matteo Figini

**Affiliations:** 1 Istituto di Sistemi e Tecnologie Industriali Intelligenti per il Manifatturiero Avanzato, Consiglio Nazionale delle Ricerche, Milan, Italy; 2 Istituto di Tecnologie Biomediche, Consiglio Nazionale delle Ricerche, Segrate, Italy; 3 Hawkes Institute and Department of Computer Science, University College London, London, United Kingdom

**Keywords:** artificial intelligence—AI, biomarker estimation, image processing, magnetic resonance imaging, quantitative MRI (qMRI)

## Introduction

Quantitative MRI (qMRI) enables the extraction of quantitative parameters from MR images that can serve as potential biomarkers. However, its clinical adoption still remains limited by practical constraints such as sequence availability, scan time, artifact sensitivity, inter-site variability, and post-processing complexity (1–3). Artificial intelligence (AI) is increasingly used to overcome these barriers throughout the workflow, from image formation to robust biomarker estimation (4–6). However, compared to other imaging applications, qMRI presents unique challenges for AI, as it's fundamental to ensure that algorithm outputs are quantitatively accurate and respect their physical meaning. This Research Topic sought contributions that address these challenges and target clinically relevant scenarios in qMRI.

A unifying theme across the Research Topic is the use of AI to make qMRI more accessible, either by extracting quantitative information from routinely acquired conventional images or by automating steps that currently require specialized expertise or are time-consuming. Specifically, two contributions focus on retrospective quantification, i.e., learning mappings from conventional acquisitions to quantitative parameters that would otherwise require specialized sequences. A third contribution targets reproducible, automated segmentation to enable fast and standardized flow quantification. Finally, one paper proposes MR fingerprinting (MRF) data synthesis from magnitude-only conventional imaging, suggesting a route to relaxometry without a dedicated MRF pulse sequence. Together, these studies illustrate complementary approaches to applying AI at various points along qMRI pipelines, such as deriving quantitative information from constrained inputs, ensuring scalability, and validating outputs against quantitative references.

## Overview of the contributions

In their paper, Sun et al. focus on retrospective T_2_ mapping of the prostate from conventional T_1_- and T_2_-weighted images, motivated by the clinical value of quantitative T_2_ for lesion characterization while acknowledging the limited availability of dedicated mapping sequences in routine mpMRI protocols. A U-Net trained against reference multi-echo spin-echo T2 maps produces estimates that preserve anatomical structure and contrast. The predicted T2 maps in 25 subjects show strong quantitative agreement with references and demonstrate clinical utility by differentiating tumor from non-tumor tissue and reflecting longitudinal changes in active surveillance cohorts. The work highlights the potential of AI to extract quantitative biomarkers from existing standard clinical imaging when rigorously validated and clinically contextualized.

Wu et al. address the limitations of standard multi-phasic abdominal DCE-MRI by proposing a pharmacokinetics-informed deep learning framework to retrospectively recover temporal resolution and enable quantitative analysis. Considering 45 subjects, including healthy controls and patients with pancreatic ductal adenocarcinoma or chronic pancreatitis, a model was trained on high-temporal-resolution DCE reference data, yielding pharmacokinetic parameters that closely match quantitative DCE estimates and discriminate healthy pancreas from disease cohorts. By constraining learning through a downstream physical model, the study exemplifies a hybrid, model-aware AI strategy well suited to quantitative MRI, enabling biomarker extraction without changes to routine imaging protocols.

Winter et al. address vessel segmentation, a key bottleneck in intracranial 4D flow MRI, by proposing a fully automated framework that reduces time burden and user variability, particularly in stenotic vessels. Using a 3D U-Net trained on dual-VENC data from 68 patients with intracranial atherosclerotic disease and stenosis and 86 healthy controls, the method achieves fast inference with accuracy comparable to expert observers. Beyond geometric performance, the study validates segmentation using downstream hemodynamic metrics, including flow parameters and flow conservation error, and observes strong agreement in lumen area with black-blood vessel wall imaging. This work demonstrates a practical and clinically relevant pathway for robust, automated extraction of flow biomarkers.

Finally, in their paper, McGee et al. propose a deep learning strategy to synthesize MRF signals from conventional magnitude-only 3D T_1_-weighted brain MRI, thereby reducing dependence on customized MRF acquisitions and dedicated processing. Using data from 37 volunteers, the authors found a high correlation between a U-Net–based model and relaxometry (T_1_, T_2_) derived from dictionary matching on synthesized vs. acquired MRF signals across 47 anatomical regions. This work is notable for framing the learning problem around quantitative endpoints (regional relaxometry agreement) rather than purely image similarity, which is essential when the intended output is a quantitative map rather than a visually plausible image.

## Outlook

Across the four contributions, several methodological priorities emerge. First, retrospective quantification from non-specialised acquisitions is an increasingly practical strategy, but it raises additional questions, since models trained on specific protocols or vendors must be extensively validated in different conditions to assess generalizability. Additionally, training and validation on retrospective data can raise concerns about how reliably the findings will translate to prospective studies, where recruitment and acquisition protocols are controlled and kept up to date. Second, AI can be used as an alternative to labour-intensive manual processing (e.g., segmentation) or computationally intensive processing (e.g., iterative algorithms for model fitting) to accelerate procedures and reduce the need for specialized expertise. Together, these two points show the potential of AI to make qMRI more accessible by reducing the need for human expertise, computational time, and specialized acquisitions or hardware, as well as improving reproducibility. However, performance assessment should be anchored in quantitative endpoints (agreement with reference maps, robustness of derived parameters, impact on downstream measurements), not solely on qualitative analysis based on visual similarity. AI applications in qMRI are most impactful when automated processing translates into clinically meaningful endpoints and when performance is explicitly validated in patient cohorts rather than extrapolated from results in healthy controls or synthetic data.

Looking forward, the direction suggested by this Research Topic is that AI will function as an enabling factor for qMRI—expanding access to quantitative biomarkers by reducing dependency on specialized sequences, accelerating analysis, and supporting more reproducible workflows. Progress toward routine use will depend on continued emphasis on external validation, transparent reporting of acquisition/preprocessing dependencies, and (where feasible) uncertainty or quality-control mechanisms that help clinicians and researchers judge when a quantitative estimate is reliable. We hope this collection will help inform both method developers and end users on practical, validation-focused pathways to translate AI-based qMRI into robust tools for research and patient care.
